# A Subset of Dogs with Presumptive Idiopathic Epilepsy Show Hippocampal Asymmetry: A Volumetric Comparison with Non-Epileptic Dogs Using MRI

**DOI:** 10.3389/fvets.2017.00183

**Published:** 2017-11-08

**Authors:** Chelsie M. Estey, Curtis W. Dewey, Mark Rishniw, David M. Lin, Jennifer Bouma, Joseph Sackman, Erica Burkland

**Affiliations:** ^1^Department of Clinical Sciences, Cornell University Hospital for Animals, Ithaca, NY, United States; ^2^Department of Biomedical Sciences, Cornell University, Ithaca, NY, United States; ^3^Rochester Veterinary Specialists, Rochester, NY, United States; ^4^Long Island Veterinary Specialists, Plainview, NY, United States

**Keywords:** epilepsy, hippocampus, MRI, dog, asymmetry

## Abstract

MRI-acquired volumetric measurements from 100 dogs with presumptive idiopathic epilepsy (IE) and 41 non-epileptic (non-IE) dogs were used to determine if hippocampal asymmetry exists in the IE as compared to the non-IE dogs. MRI databases from three institutions were searched for dogs that underwent MRI of the brain and were determined to have IE and those that were considered non-IE dogs. Volumes of the right and left hippocampi were measured using Mimics^®^ software. Median hippocampal volumes of IE and non-IE dogs were 0.47 and 0.53 cm^3^, respectively. There was no significant difference in overall hippocampal volume between IE and non-IE dogs; however, IE dogs had greater hippocampal asymmetry than non-IE dogs (*P* < 0.012). A threshold value of 1.16 from the hippocampal ratio had an 85% specificity for identifying IE-associated asymmetry. Thirty five percent of IE dogs had a hippocampal ratio >1.16. Asymmetry was not associated with any particular hemisphere (*P* = 0.67). Our study indicates that hippocampal asymmetry occurs in a subset of dogs with presumptive idiopathic/genetic epilepsy, suggesting a structural etiology to some cases of IE.

## Introduction

Idiopathic epilepsy (IE) is the most commonly diagnosed canine neurologic disorder in veterinary medicine (1). Almost half of all dogs presenting for seizures are subsequently diagnosed with IE, and in one study IE was diagnosed in 75% of dogs with an onset of seizures before 1 year of age (2, 3). Approximately 25–30% of dogs treated for IE are refractory to treatment or are poorly controlled, often requiring multiple anticonvulsant drugs (4). Moreover, epileptic dogs that exhibit cluster seizures or status epilepticus have shorter lifespans than those that do not experience these episodes (3, 5, 6). Finally, many breeds are susceptible to IE, but how epilepsy in different breeds differs is poorly understood (4).

Our understanding of epilepsy in dogs, and how many forms of epilepsy might exist, is still rudimentary. Consequently, our inability to accurately diagnose the type of epilepsy in affected dogs likely hampers our ability to optimally treat these patients. Ion channels, particularly calcium and sodium channels, play an important role in maintaining cellular and electrical homeostasis in the brain. Mutations in genes encoding these ion channels result in epilepsy in humans by causing ion channel dysfunction (7–9). It is suspected that similar channelopathies exist in epileptic dogs, and the majority of anticonvulsant drugs used in humans and dogs have mechanisms of action involving ion channels (4). However, despite advances in anticonvulsant medications over the years, a substantial subset of epileptic dogs (between 25 and 30%) are refractory to these drugs (4).

In humans, a population of epileptics with focal abnormalities of brain architecture has been well-documented. These patients have what is often referred to as localization-related epilepsy, the most common example of which is temporal lobe epilepsy, particularly mesial temporal lobe epilepsy (mTLE) (10, 11). mTLE is the most common type of localization-related epilepsy in people (10, 12) and is characterized by hippocampal sclerosis (HS), due to atrophy of the hippocampal gray matter (13, 14). Characteristic histopathological features of HS include neuronal loss, astrogliosis, granule cell dispersion, and mossy fiber sprouting (15, 16). Although imaging and pathological features of mTLE and HS have been described in some detail in people, the etiology of this subset of epileptics is not entirely clear and likely represents a number of different abnormalities with a similar phenotypic expression (15, 16). Overall, it has not been definitively determined if the anatomic and histopathologic abnormalities that typify mTLE/HS are a cause or a result of excessive seizure activity, or some combination thereof (15, 16). Multiple causative factors have been implicated in the development of human mTLE/HS, including febrile seizures, brain trauma, inflammatory brain conditions, as well as genetic and epigenetic factors; it is likely that many cases may be multifactorial with regard to cause (15–19). Regardless of cause, the abnormal hippocampus is structurally and functionally connected to the adjacent mesial temporal lobe, and these structures are believed to develop an epileptic circuit (20). MRI can detect focal changes in brain architecture that can provide clues regarding etiology and treatment; this is visible as a change in the volume of a given region of the brain (14). The prevalence of mTLE/HS is high enough that automated quantitative measurement techniques have been developed to detect hippocampal asymmetry (21). This identification has important treatment implications, as patients with mTLE with HS are often refractory to anticonvulsant drug therapy (10). Despite this drug refractoriness, surgical resection of the seizure focus (temporal lobe with or without hippocampal tissue) has a very high success rate in mTLE/HS people who are refractory to drug therapy (12, 22–27).

Currently, minimal information exists regarding location-dependent changes observed on MRIs from epileptic dogs (28). If a population of dogs with location-dependent epilepsy exists, surgical intervention could offer an alternative treatment for effective management of this type of epilepsy. Therefore, we sought to characterize hippocampal asymmetry in a large cohort of dogs with IE using volumetric imaging software and to compare asymmetry in this population with non-IE dogs. Furthermore, we sought to identify a subset of dogs with IE that might demonstrate substantial hippocampal asymmetry.

## Materials and Methods

MRI databases from three institutions (Cornell University Hospital for Animals, Long Island Veterinary Specialists, and Rochester Veterinary Specialists) were searched for dogs that underwent MRI of the brain and were determined to have IE and those that were considered non-IE dogs. The diagnosis of IE was based on characteristic clinical features of the disorder (i.e., recurrent seizures with normal neurological examination interictally and normal bloodwork), combined with normal MRI results. The non-IE group consisted of dogs that were imaged for ear disease, nasal disease, vestibular disease, and cranial nerve deficits. Medical records were reviewed and animals were excluded from the study if their clinical signs did not fit with a presumptive diagnosis of IE or had a multifocal neurolocalization. Animals were excluded from the non-epileptic (non-IE) group if they had centralizing neurologic signs or had any intracranial lesions on MRI.

All MRIs were performed under general anesthesia with either a 1.0 T (Siemens Magnetom Harmony, 1.0 T, Munich, Germany), 1.5 T (Philips Achieva, 3.0 T, NJ, USA), or 3.0 T unit (Toshiba Vantage Elan, 1.5 T, CA, USA). Imaging sequences were obtained in transverse (T2 fluid-attenuated inversion recovery (FLAIR), T2- and T1-weighted, and T1-weighted post-gadolinium sequences), sagittal (T2- and T1-weighted post-gadolinium sequences), and dorsal planes (T1-weighted post-gadolinium sequence). For the 1.0 and 1.5-T MRI units, measurement parameters were as follows: slice thickness, 3.5 mm; slice gap, 3.5 mm; FOV, 185 mm; matrix size of images, 480 × 480. For the 3.0-T MRI unit, measurement parameters were as follows: slice thickness, 2.0 mm; slice gap, 1.0–3.0 mm (depending on dog size); FOV, 1101 mm; matrix size of images, 400 × 400.

**Figure 1:**
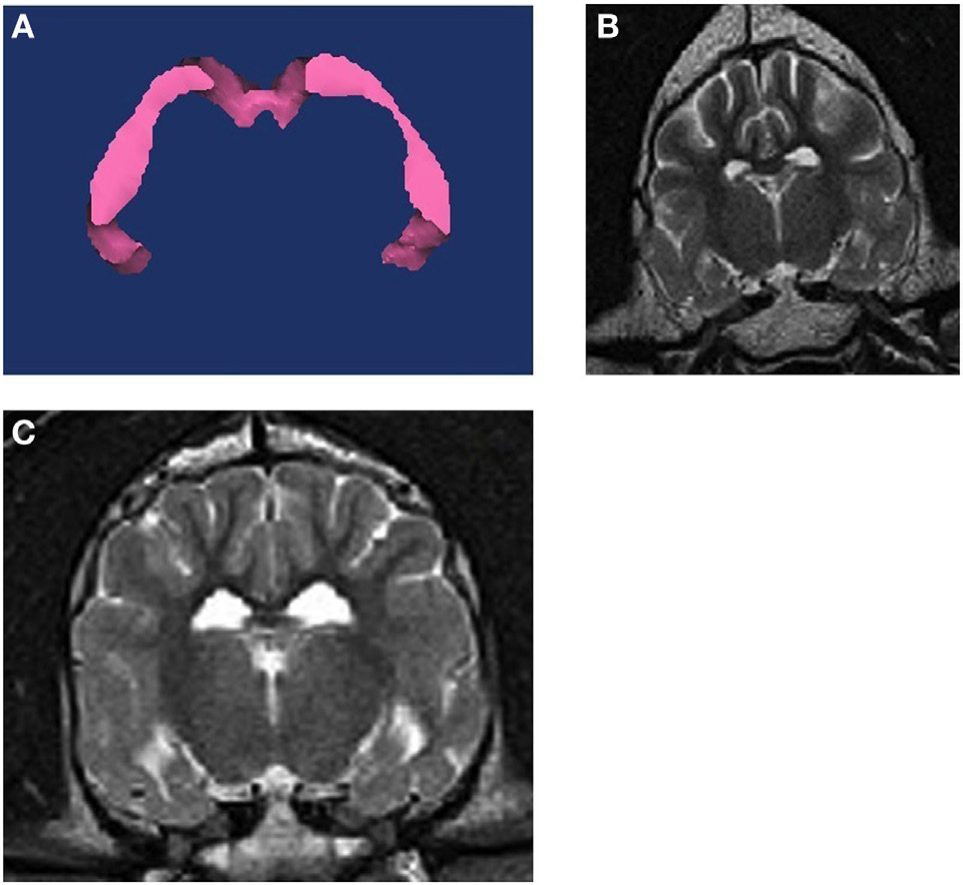
**(A)** 3D volumetric analysis of the hippocampus. **(B)** T2W transverse MRI image at the level of the hippocampus from a non-epileptic dog. **(C)** T2W transverse MRI image at the level of the hippocampus from an epileptic dog. The right hippocampus and temporal lobe region is notably smaller on the right side of the brain.

For each dog, three-dimensional volumes were measured using Mimics^®^ software by one observer (Joseph Sackman) who was unaware of the status of the dogs in the study. Quantitative volumetric measurements comparing the right and left hippocampi were made for each T2-weighted slice to obtain the full anatomic boundaries these structures (Figure 1). The anatomic landmarks for the measurements were used from published reference information (29, 30). Each structure was measured rostral to caudal using a technique of selecting the specific pixels associated with that structure per slice. Within each slice, pixels are painted with a selection tool that distinguishes them from the rest of the image. Once all pixels are selected in each slice, the software can then render a three-dimensional representation of the structure. Volume data are then derived from this representation. We used a simple body-size-independent measure of hippocampal asymmetry by obtaining the ratio of the larger hippocampal volume to the smaller hippocampal volume. Perfect symmetry would result in a ratio of 1.0, with increasing asymmetry being represented by values increasingly greater than 1.0. Because previous investigators had used a normalization technique (absolute difference divided by the greater of the two hippocampi), we also evaluated our data using this method (28).

Descriptive statistics were provided for estimates of hippocampal volume differences for both IE and non-IE dogs. Data were examined for normality by a Shapiro–Wilk test for each group. Because the data were not normally distributed, comparisons of hippocampal asymmetry between groups were performed using a Mann–Whitney *U* test. To determine if asymmetry in dogs with IE was biased to one side or the other, we compared the proportion of asymmetry on the right side using a *z*-test against a nominal value of 0.5. A *P*–value <0.05 was considered significant. Receiver operating characteristic analysis was used to identify a threshold for hippocampal asymmetry that would reliably separate dogs with hippocampal asymmetry from dogs without hippocampal asymmetry. We then used this threshold value to examine the specificity of identifying epilepsy-associated asymmetry.

To examine repeatability of MRI measurements, 20 patient scans were randomly selected (using a random number generator) and all variables were re-measured by the single observer (Joseph Sackman), who was blinded to the previous measurements for the cases. Agreement between repeated measurements was examined using Limits of Agreement analysis (31).

## Results

We included 100 dogs with IE and 41 non-IE dogs in the study. The median age of the IE dogs was 60 months and that of the non-IE dogs was 86 months. Non-IE dogs were older than IE dogs at the time of MRI (*P* = 0.01), but had similar distributions of sex (44% male and 56% female in each group) and proportions of purebred dogs (75 vs 82%).

**Figure 2:**
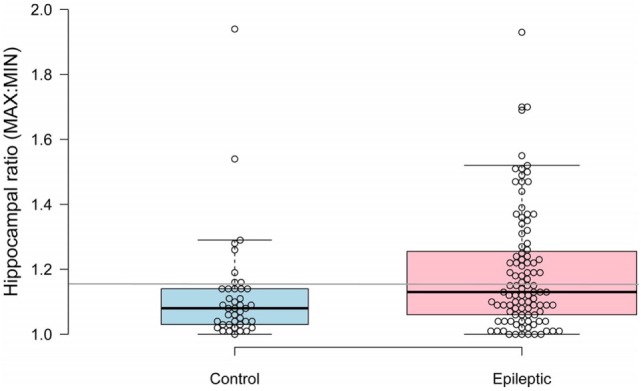
Scatterplot comparing hippocampal ratios between non-epileptic and epileptic dogs. A threshold value of 1.16 (denoted by gray horizontal line) had an 85% sensitivity for identifying idiopathic epilepsy-associated asymmetry.

Median hippocampal volumes of IE and non-IE dogs were 0.47 and 0.53 cm^3^, respectively. There was no significant difference in overall hippocampal volumes between the two groups (*P* = 0.117). Epileptic dogs had greater hippocampal ratios than non-IE dogs (*P* = 0.012, Figure 1). The ROC analysis identified a threshold hippocampal ratio of 1.14 as the optimal discriminating value. Using this threshold value, 47% of IE dogs and 20% of non-IE dogs would be considered to have asymmetry, resulting in a specificity for identifying epilepsy-associated hippocampal asymmetry of 85%. To slightly improve the specificity, we increased the threshold hippocampal ratio to 1.16. Using this higher ratio, 42% of IE dogs and 15% of non-IE dogs would be considered to have hippocampal asymmetry (*P* = 0.002), resulting in a specificity for identifying epilepsy-associated hippocampal asymmetry of 87% (Figure 2.). Hippocampal asymmetry was not biased to one hemisphere (*P* = 0.8).

Epileptic dogs had greater normalized hippocampal differences than non-IE dogs (median normalized difference 11.6 vs 7.1%). Using the historical threshold of 6% to denote asymmetry (15), 73% of IE dogs and 58% of non-IE dogs would be considered to have hippocampal asymmetry, resulting in 75% specificity for identifying epilepsy-associated hippocampal asymmetry. Conversely, using a normalized hippocampal difference >12.6% to denote asymmetry, 48% of IE dogs, and 20% of non-IE dogs would be considered to have hippocampal asymmetry, resulting in 86% specificity for identifying epilepsy-associated hippocampal asymmetry.

The observer performing the measurements of brain volume showed measurement repeatability for total hippocampal volumes to within approximately 40 voxels, representing a median% difference of 5.2% for the 20 randomly selected cases, without noticeable fixed or proportional bias.

## Discussion

Our study shows that dogs with IE have greater hippocampal asymmetry than non-IE dogs, and that a greater proportion of epileptic than non-IE dogs have hippocampal ratios indicative of asymmetry using criteria similar to those in humans. The lack of significant difference between IE and non-IE groups with regard to overall hippocampal volume emphasizes the utility of analyzing hippocampal asymmetry using ratios vs total volumes. Whether the subset of epileptic dogs with hippocampal asymmetry (approximately 40%) has particular clinical or pathological characteristics (e.g., increased refractoriness to anticonvulsant therapy, temporal lobe epilepsy) remains to be determined. Due to the retrospective nature of this study, the authors were unable to obtain accurate clinical data regarding seizure control for the majority of IE dogs; because of this limitation, it was decided to focus the current study only on volumetric imaging and to pursue possible correlates between hippocampal asymmetry and drug refractoriness in IE dogs in a future prospective investigation.

Our findings are similar to but expand on those of previous investigators (28). Using similar methods to identify asymmetry as those investigators, our data differ in terms of the magnitude of asymmetry in IE and non-IE dogs and in terms of proportions of dogs with asymmetry in each group. Our data suggest that a threshold value of 6% for a normalized difference is likely too small to accurately identify hippocampal asymmetry and that a value of 12.6% more accurately identifies IE dogs. Similarly, we would propose a hippocampal ratio of 1.16–1.2 to define hippocampal asymmetry in epileptic dogs.

Hippocampal sclerosis is the most common neuropathological change encountered in human patients with mTLE. The causative factors of mTLE/HS have not been fully elucidated, and there are believed to be a number of subtypes of this disorder, with different etiologies (15, 16). Human patients affected by TLE/HS often present with seizures refractory to drug therapy, which are often amenable to surgical resection of the epileptogenic focus. Hippocampal asymmetry, resulting from unilateral HS, is present in 60–70% of patients with mTLE who pursue surgical treatment of refractory seizures (21). In these refractory patients, surgical treatment is associated with abolition of seizures or a significant reduction in seizure frequency in the majority of these patients (32–35) Therefore, the identification of hippocampal asymmetry in IE dogs, especially those refractory to anticonvulsant therapy, might encourage neurosurgeons to consider resection of the temporal lobe on the affected side. Because radical cerebral resection can be performed in dogs with minimal lasting effects (36), surgical intervention in these patients, if successful, could provide an alternative to life-long anticonvulsant therapy, poor seizure control, and the attendant emotional and financial impacts on the owner. As is the case with human epilepsy, poor seizure control and adverse effects of anticonvulsant medication have been shown to be significantly correlated with poor quality of life in dogs with IE, as perceived by owners (37–39). In addition, the negative impacts of IE on the daily life of epileptic dog owners have been documented (40). Whether this subset of dogs with hippocampal asymmetry would benefit from surgical intervention remains speculative at present. Additional histopathological studies of this subset of dogs, to confirm that HS is in fact present, are warranted. In addition, prospective investigation into the nature of seizure activity (i.e., refractory vs non-refractory) in dogs with hippocampal asymmetry is necessary. Further study into the nature of canine mTLE/HS may also help to elucidate causative factors of the condition. Specifically, considering that genetic and epigenetic factors have been implicated in human mTLE/HS, studying individual canine breeds that are prone to refractory epilepsy (and demonstrate hippocampal asymmetry) may lead to discovery of causative genetic disorders and/or molecular mechanisms of disease development.

Our study sets the stage for further evaluation of a subset of dogs with IE that appear to have hippocampal asymmetry. Future prospective imaging studies are planned in order to further investigate more detailed features of hippocampal and temporal lobe differences in dogs with hippocampal asymmetry, using advanced MR sequences such as tractography. If our hypothesis, that these dogs might have surgically amenable location-specific epileptogenic foci, is correct, seizure management in a sizeable proportion of epileptic dogs might take a large step forward.

## Author’s Note

Presented in abstract form at the 2014 American College of Veterinary Radiology Annual Scientific Meeting, St. Louis, Missouri, October 2014.

## Author Contributions

CD—inception and supervision of project, involved in case collection, and manuscript preparation. CE—organization and collating data, writing manuscript, and literature search (resident project). MR—data analysis, statistics, manuscript review, and revision. JS—data collection, volumetric measurements, collating and organizing data. JB—evaluating MR images, manuscript preparation, and review. DL—involved in inception and supervision of project, and manuscript review. EB—assisted in data collection and organization, and manuscript review.

## Conflict of Interest Statement

The authors declared no potential conflicts of interest with respect to the research, authorship, and/or publication of this article. The reviewer RP and handling editor declared their shared affiliation.
